# Peg-Grafted Liposomes for L-Asparaginase Encapsulation

**DOI:** 10.3390/pharmaceutics14091819

**Published:** 2022-08-29

**Authors:** Marina de Souza Guimarães, Jorge Javier Muso Cachumba, Cecilia Zorzi Bueno, Karin Mariana Torres-Obreque, Grace Verónica Ruiz Lara, Gisele Monteiro, Leandro Ramos Souza Barbosa, Adalberto Pessoa, Carlota de Oliveira Rangel-Yagui

**Affiliations:** 1 Department of Biochemical and Pharmaceutical Technology, School of Pharmaceutical Sciences, University of São Paulo, São Paulo 05508-000, SP, Brazil; 2 Department of General Physics, Institute of Physics, University of São Paulo, São Paulo 05508-000, SP, Brazil; 3 Brazilian Synchrotron Light Laboratory (LNLS), Brazilian Center for Research in Energy and Materials (CNPEM), Campinas 13083-100, SP, Brazil

**Keywords:** liposome, pegylated liposome, nanoreactor, nanocarrier, L-asparaginase, acute lymphoblastic leukemia

## Abstract

L-asparaginase (ASNase) is an important biological drug used to treat Acute Lymphoblastic Leukemia (ALL). It catalyzes the hydrolysis of L-asparagine (Asn) in the bloodstream and, since ALL cells cannot synthesize Asn, protein synthesis is impaired leading to apoptosis. Despite its therapeutic importance, ASNase treatment is associated to side effects, mainly hypersensitivity and immunogenicity. Furthermore, degradation by plasma proteases and immunogenicity shortens the enzyme half-life. Encapsulation of ASNase in liposomes, nanostructures formed by the self-aggregation of phospholipids, is an attractive alternative to protect the enzyme from plasma proteases and enhance pharmacokinetics profile. In addition, PEGylation might prolong the in vivo circulation of liposomes owing to the spherical shielding conferred by the polyethylene (PEG) corona around the nanostructures. In this paper, ASNase was encapsulated in liposomal formulations composed by 1,2-dioleoyl-sn-glycero-3-phosphocholine (DOPC) or 1,2-dimyristoyl-sn-glycero-3-phosphocholine (DMPC) containing or not different concentrations of 1,2-distearoyl-sn-glycero-3-phosphoethanolamine-N [methoxy (polyethylene glycol)-2000] (DSPE-PEG). Nanostructures of approximately 142–202 nm of diameter and polydispersity index (PDI) of 0.069 to 0.190 were obtained and the vesicular shape confirmed by Transmission Electron Microscopy (TEM and cryo-TEM). The encapsulation efficiency (%EE) varied from 10% to 16%. All formulations presented activity in contact with ASNase substrate, indicating the liposomes permeability to Asn and/or enzyme adsorption at the nanostructures’ surface; the highest activity was observed for DMPC/DSPE-PEG 10%. Finally, we investigated the activity against the Molt 4 leukemic cell line and found a lower IC_50_ for the DMPC/DSPE-PEG 10% formulation in comparison to the free enzyme, indicating our system could provide in vivo activity while protecting the enzyme from immune system recognition and proteases degradation.

## 1. Introduction

Acute Lymphoblastic leukemia (ALL) is a cancer of the blood and bone marrow that affects mostly children and adolescents. It is characterized by abnormal proliferation and differentiation of clonal lymphocytes that causes anemia, thrombocytopenia, leukopenia and granulocytopenia [1]. Treatment includes the enzyme L-asparaginase (ASNase), which catalyzes the hydrolysis of L-asparagine (Asn) in bloodstream resulting on the products aspartic acid (Asp) and ammonia (NH_3_) [2]. While normal cells produce Asn from L-aspartate with the help of asparagine synthetase, leukemic cells lack this enzyme and without Asn protein synthesis is impaired, resulting in apoptosis [3]. Since its introduction in therapeutic protocols, in 1970, the survival rates increased from 5% to approximately 90% [4]. Nonetheless, as a protein drug, ASNase, presents pharmacokinetic limitations, such as short therapeutic half-lives, plasma instability and immunogenicity [5,6]. Due to its bacterial origin, the degradation of ASNase by blood proteases is considerable and the epitopes generated are easily recognized by the immune system, promoting an immune response [7]. One way to tackle this problem refers to ASNase encapsulation in nanostructures such as liposomes [8,9].

Liposomes are composed of amphiphilic molecules (phospholipids) that in aqueous media self-assemble in bilayers surrounding an aqueous core [10]. The benefits of liposomes as nanocarriers are well known: they can modulate pharmacodynamics and pharmacokinetics of encapsulated drugs [8,9,11], protect against biodegradation, enhance solubility, decrease chemical degradation and decrease toxicity [12]. Nonetheless, the rapid clearance of conventional liposomes owing to recognition by the Reticule Endothelial System (RES) led to the development of stealth liposomes, i.e., coated with polymeric molecules like polyethylene glycol [13]. PEGylated liposomes show higher stability and longer half-life in blood due to reduced capture by RES [14]. An example of successful formulation is Doxil^®^, doxorubicin in PEGylated liposomes [15,16]. It is worth mentioning not only pegylated nanostructures have been investigated to improve protein drugs circulation time, but protein-PEG bioconjugation is also a common strategy [17], as well as other polymer bioconjugation have recently been proposed [18,19]. Nonetheless, PEG itself has the potential to induce an immune response, generating anti-PEG antibodies. However, despite several studies showing that anti-PEG antibodies were responsible for an attenuated response for pegylated protein drugs, few studies have investigated whether they significantly influence the pharmacokinetics of the proteins. In a recent work, Grenier et al. (2018) showed that the PEG effect on in vivo clearance varies among different types of nanostructure. More specifically, they found it was much more pronounced for PEG-PLGA nanoparticles (2.9-fold) than for pegylated liposomes (1.5-fold) [20].

Liposomes and their polymeric counterpart, polymersomes, have also been investigated as biocatalytic nanoreactors. Depending on the composition, the phospholipid or polymeric membrane can encapsulate the enzyme and at the same time allow small MW substrates translocation, so the aqueous core provides a separate compartment for the enzymatic reaction, similar to cells and organelles [21,22]. For liposomes, one can modulate the bilayer permeability based on the phospholipids compositions, variating the gel-to-fluid phase transition temperature of the phospholipids (T_m_) or the pH [21,22,23]. As a matter of fact, our group has recently proposed asymmetric polymersomes of PMPC_25_-PDPA_70_/PEO16-PBO_22_ for ASNase nanoencapsulation and showed it was permeable to the substrate [24]. Nonetheless, we could not test in vitro activity against leukemic cells, yet. In addition, this is an initial proof of concept of a polymer-based nanostructure with a polymer that is relatively new and not approved by the regulatory agencies (FDA and EMA). As much as we consider it promising, we know it would take a while to have something like this available, reason why we decided to investigate classic liposomes.

In a previous work, an ASNase-loaded conventional liposome formulation was developed; however, in vitro activity against leukemia cell lines was not determined [25,26]. Moreover, other related papers describe systems with significant variations in encapsulation efficiency (from 20% up to 72%) and no clear correlation with enzyme activity [25,26,27]. Other concern that motivated this work is, as mentioned before, conventional liposomes are easily recognized by the RES and present rapid clearance. Here, we produced PEGylated liposomes for ASNase encapsulation that proved to be capable of Asn depletion and presented in vitro activity against leukemic cells (Molt 4); therefore, we present a novel formulation with strong potential to improve ASNase based treatment in a short period of time.

## 2. Materials and Methods

### 2.1. Materials

Phospholipids 1,2-dioleoyl-sn-glycero-3-phosphocholine (DOPC), 1,2-dimyristoyl-sn-glycero-3-phosphocholine (DMPC) and 1,2-distearoyl-sn-glycero-3-phosphoethanolamine-*N*- [methoxy (polyethylene glycol)-2000] (DSPE-PEG) were purchased from Avanti Polar Lipids^®^ Inc. (Alabaster, AL, USA). Asparaginase (ASNase) was purchased from ProSpec Tany (Rehovot, Israel), and α-tocopherol was from Sigma-Aldrich Chemical Co. (St. Louis, MO, USA). Ultra-purified or distilled water were used to prepare the solutions. All other reagents used were of analytical grade or HPLC grade when required.

### 2.2. Liposomes Preparation

Two types of phospholipids were used to prepare the liposomal formulations, namely the saturated DMPC and the unsaturated DOPC. For the pegylated liposomes, different concentrations of DSPE-PEG were added (5% or 10% molar). In addition, 1% (molar) of α-tocopherol was added in the DOPC formulations to avoid oxidation. Liposomal formulations were prepared by thin film hydration [28]. Briefly, the phospholipids were solubilized in previously filtrated chloroform and the final solution concentration was 5 mg/mL. The solutions were dried for 1 h under reduced pressure (R-100 rotary evaporator, Büchi, Flawil, Switzerland) to form a thin film that was further hydrated with saline phosphate buffer (PBS) (pH 7.4) for 10 min in cycles of 1-min vortex (Vortex Mixer, VIXAR, Berlin, Germany) and 1-min water bath at 37 °C. The systems were extruded 15-times through 0.2 µm polycarbonate membranes housed in a hand-held mini-extruder (Avanti Polar Lipids^®^ Inc., Alabaster, AL, USA) at 37 °C.

### 2.3. Dynamic Light Scattering (DLS), Zeta Potential (ζ) and Stability Analysis

The hydrodynamic diameter (z-average) and Zeta potential values of the formulations were determined using NanoZS 90 Zetasizer (Malvern Instruments, Worcestershire, UK) at a temperature of 25 °C. For DLS analysis, samples of the liposomal systems were diluted 10 times in PBS. After equilibration time (60 s), three cycles of measurement with a 90° scattering angle were conducted for each analysis and mean size (z-average) and polydispersity index (PDI) were recorded. For the Zeta potential measurements, samples were diluted five times in ultra-pure Milli-Q water or PBS and three cycles of measurements were conducted for each analysis.

### 2.4. Asparaginase Encapsulation

A 5 mg/mL ASNase solution in PBS (pH 7.4) was mixed with an equal amount (400 µL) of a pre-assembled liposome dispersion. The mixture was transferred to a 4 mm electroporation cuvette (BioRad, Hercules, CA, EUA) and electroporated in a Gene Pulser Xcell (BioRad, Hercules, CA, EUA) at voltage of 1000 V, using 10 pulses at 60-s intervals. Size exclusion chromatography (SEC) was used to remove the non-encapsulated ASNase [29]. Briefly, 800 µL of each liposomal formulation was passed through a Sephadex 4B column using PBS (pH 7.4) as eluent. Eluted fractions were collected and evaluated by DLS to confirm the presence of nanostructures.

### 2.5. Determination of Encapsulation Efficiency

Size Exclusion Chromatography was performed to indirectly determine the ASNase encapsulation efficiency in liposomes formulations based on a protocol adapted from Bartenstein and collaborators [30]. ASNase-loaded liposomal systems without purification, i.e., containing encapsulated enzyme as well as nonencapsulated enzyme, were injected into a Superdex 200 Increase 0/300 GL column (GE Healthcare Life Science, Uppsala, Sweden) and the elution was performed isocractically in a Fast Protein Liquid Chromatography (FPLC) ÄKTA Purifier (GE Healthcare Life Sciences, Uppsala, Sweden) in PBS (pH 7.4) with 0.5 mL/min flow rate. The absorbance was monitored at 280 nm and protein concentration calculated by integrating the peak corresponding to the free protein (*P_non−encapsulated_*) and the peak corresponding to the initial solution of enzyme (*P_i_*). The EE% was calculated according to the Equation (1):

(1) $$\%EE=\frac{\left(P_{i}-P_{non-encapsulated}\right)}{P_{i}}\times 100$$

### 2.6. Transmission Electron Microscopy Analysis

Transmission electron microscopy (TEM) was carried out in a Jeol 100 CX II microscope at an acceleration voltage of 80 kV to characterize the morphological structure of liposomes (shape and size). Carbon grids of Formvar/Carbon 300 Mesh (Electron Microscopy Sciences, Hatfield, UK) were previously submitted to a Glow Discharge Cleaning System process (PELCO easyGlow™, Redding, CA, USA) (30 mA for 45 s) under reduced pressure, then 5 µL of sample was pipetted onto the grid and left for 1 min. Sample excess was carefully dried with filter paper, 20 µL uranyl acetate was added and left for 1 min; the excess was removed with filter paper. The grids were dried at room temperature for 24 h. Cryo-EM measurements of the liposomes were performed in a Talos Arctica (Thermo Fisher, Waltham, MA, USA) at 200 kV with a CMOS camera OneView 4 k × 4 k (Stemmer Imaging, Graz, Austria) at the the Brazilian National Laboratory of Nanotechnology (LNNano, Campinas-São Paulo, Brazil). A 300 mesh Holey Lacey Carbon from Ted Pella^®^ was previously submitted to a glow discharge Cleaning System (15 mA, 10 s) prior to the drop casting of the sample. Samples were vitrified in a Vitrobot^®^ using liquid ethane. Finally, grids were then transposed to a 12-spaces grid box in liquid nitrogen until measurements. The images were processed using the Digital Micrograph^TM^ software (Pleasanton, CA, USA). ImageJ was used to analyze liposome size and membrane thickness.

### 2.7. Liposomes Stability over the Time

The stability of the liposomal formulations at 4 °C and at 37 °C throughout a 40 days period was investigated based on particle size distribution and polydispersity [31].

### 2.8. Liposome Permeability Assay

Permeability to L-Asn was investigated by measuring ASNase activity in ASNase-loaded liposomes over the time, using the Nessler method, a colorimetric assay that measures ammonium released from L-Asn hydrolysis by ASNase [32]. Briefly, 50 µL of PBS (pH 7.4), 200 µL of ultrapure water and 50 µL of asparagine (25 mg/mL) were mixed and incubated at 37 °C for 5 min. Then, 50 µL of the purified ASNase-loaded liposomes formulation was added and the mixture was incubated at 37 °C for 30, 60 or 120 min. Following, 50 µL of 1.5 M trichloroacetic acid (TCA) was added to stop the enzymatic reaction. A 100 µL sample was diluted 5 times with ultrapure water and 250 µL of Nessler reagent was added. After 1 min of reaction, a 200 µL sample was transferred to a 96 well plate and absorbance measured at 436 nm using a spectrophotometer SpectraMax Plus 384 (Molecular Devices, San Jose, CA, USA). The enzyme activity was calculated based on a (NH_4_)_2_SO_4_ calibration curve, considering that one unit of ASNase (U) catalyzes the formation of 1 µmol of ammonia per minute at 37 °C. The experiments were carried out in triplicates. As a control, 5 mg/mL ASNase solution in PBS (pH 7.4) was mixed with an equal amount (400 µL) of a pre-assembled liposome dispersion and submitted to the same protocol, however without performing the electroporation.

### 2.9. In Vitro Cytotoxicity against MOLT-4 Cells

MOLT-4 cells previously frozen in liquid nitrogen were activated in Roswell Park Memorial Institute medium (RPMI 1640) supplemented with 10% of fetal bovine serum, 2.5 g/L of glucose, 0.01 M of HEPES and 1 mM of sodium pyruvate. Cells were pealed after reaching a high cell density with confluence greater than 90%. After reaching confluence, cell lineages were centrifuged at 600 xg at 4°C for 10 min and suspended in fresh RPMI medium. Cell viability was visualized with Trypan blue (Sigma-Aldrich, Darmstadt, Hessen, Germany) and cells were counted in Neubauer’s chamber. MTT assay was performed with the formulations ASNase-DMPC and ASNase-DMPC/DSPE-PEG 10% as well as with empty liposomes, as a control. Free enzyme was also used to compare the results. All samples (formulations and free enzyme solution) were sterilized by filtration in sterile filter Millex 13 mm PVDF 0.22 µm.

For the MTT test, 96-well cell culture-treated flat-bottom microplate was used for incubating 2 × 10^4^ cells/well with the same RPMI medium used to activate the cells. MOLT-4 cells were then treated with 0, 0.000088, 0.00032, 0.00088, 0.002, 0.004 and 0.0081 U/mL of all formulations and free enzyme and incubated for 72 h at 37 °C and 5% of CO_2_. After incubation period, 0.5 mg/mL of MTT was added in each well and incubated again for 3 h at 37 °C and 5% of CO_2_. To precipitate the formazan crystals, the microplate was centrifuged at 280 g and room temperature for 10 min and the supernatant was discarded. Finally, 200 µL of DMSO 100% was added and incubated for 5 min at 37 °C before reading the absorbance at 570 nm. Experiments were performed in triplicate. Pure DMSO and PBS 1× were used as a positive and negative controls and IC_50_ values were calculated for each formulation.

### 2.10. Statistical Analysis

Differences between the experimental groups were analyzed by Two-way ANOVA followed by Tukey’s tests, GraphPad Prism were used. Results are expressed as the mean ± standard deviation (SD) or standard error (SEM). Groups were compared considering 95% confidence interval.

## 3. Results and Discussion

### 3.1. Liposomes Preparation and Characterization

**Figure 1:**
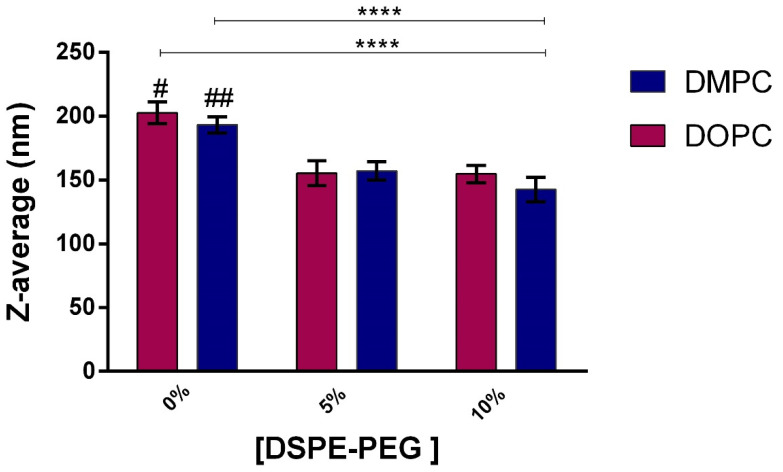
Dynamic light scattering profile of liposome formulations. Z-average values for increasing concentrations of DSPE-PEG. # formulations showed significant difference compared to PEGylated liposomes. #, ## formulations showed significant difference compared to DOPC/DSPE-PEG 5% and DMPC/DSPE-PEG 5%, respectively. Data are presented as mean ± SD (*n* = 3 independent experiments), (Two-way ANOVA, α = 0.05, **** *p* < 0.0001, Turkey test).

DLS measurements, indicate that liposome formulations of both pure DOPC or DMPC presented monomodal distributions (Figures S1 and S4) and Z-average of 202 nm and 193 nm, respectively, in agreement with the literature [33,34]. Pegylated liposomes were smaller; mean Z-average 142–157 nm (Table 1, Figures S2, S3, S5 and S6). A significant decrease in hydrodynamic diameter was observed for the systems with 5 % or 10 % of DSPE-PEG (Table 1 and Figure 1); Z-average results confirm the influence of the pegylated lipid on particle size, as described by other authors [31,35], which can be related to two factors: (i) low solubility of DSPE-PEG 2000 in lipid bilayers (4 to 10 mol%) [36,37,38], or (ii) different types of PEG conformation (mushroom or brush). More specifically, at low concentrations the polymer tends to assume a mushroom-like configuration (the polymer headgroups do not interact), leading to a compact bilayer. Upon increasing concentration, the polymer headgroups of PEGylated phospholipids assume an extended brush configuration and the steric interactions lead to a rise in lateral pressure that tend to expand the lipid membrane. With further increase in the concentration of PEGylated phospholipids, they tend to disperse in aqueous solution and induce micelle formation [39]. Literature reports that the hydrodynamic size of liposomes as well as PEGylation influences its circulation time in the bloodstream [40]. An in vivo study reported longer circulation times of PEGylated liposomes in mice in comparison to conventional liposomes [40], and liposomes of 70–200 nm were described as long-circulating [41]. Regarding polydispersity, all six formulations presented PDI values < 0.2 and were considered monodisperse [42].

The formulations reported here are based on zwitterionic and PEGylated phospholipids; therefore, charge should not play a major role in colloidal stabilization. Nonetheless, zeta potential was measured in PBS but only one measurement for each formulation due to the cuvette deterioration in the presence of PBS [43]. As showed in Table 1, irrespective of the DSPE-PEG presence, all formulations presented slightly negative zeta potential values similar to the ones previously reported in the literature for DMPC and DOPC liposomes at similar conditions, which present values between −2 and −6 mV [33,43,44]. As a matter of fact, some authors refers to liposomes with zeta potential values from −5 mV to +5 mV as neutrals [45].

### 3.2. Liposomes Stability

**Figure 2:**
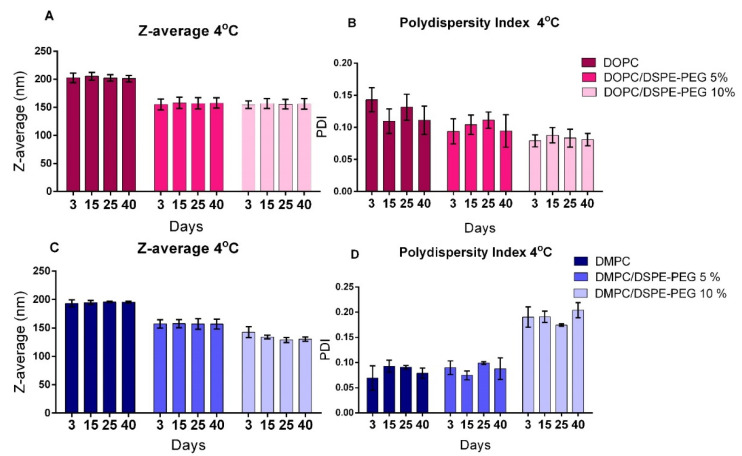
Physical stability of liposomal formulations stored at 4 °C. (**A**,**B**): Z-average and PDI, respectively, of DOPC, DOPC/DSPE-PEG 5% and DOPC/DSPE-PEG 10% formulations; (**C**,**D**): Z-average and PDI, respectively, of DMPC, DMPC/DSPE-PEG 5% and DMPC/DSPE-PEG 10% formulations. *n* = 3 independent experiments. Mean ± SD.

**Figure 3:**
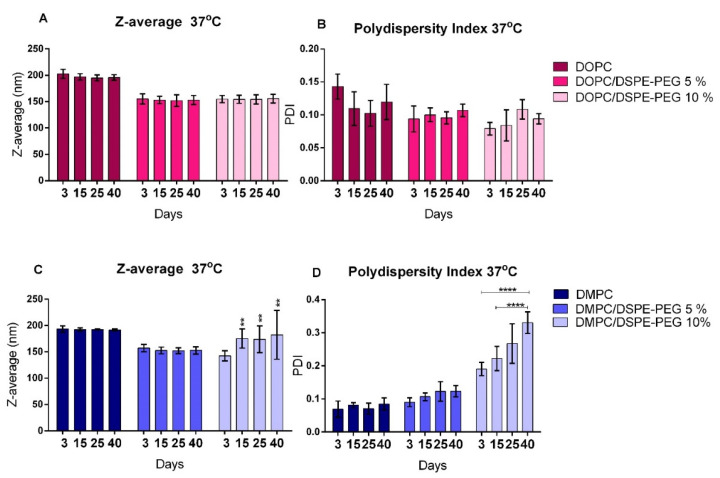
Physical stability analysis of liposomal formulations stored at 37 °C. (**A**,**B**): Z-average and PDI, respectively, of DOPC, DOPC/DSPE-PEG 5% and DOPC/DSPE-PEG 10% formulations; (**C**,**D**): Z-average and PDI, respectively, of DMPC, DMPC/DSPE-PEG 5% and DMPC/DSPE-PEG 10% formulations. *n* = 3 independent experiments. Mean ± SD, Tukey’s test, ** *p* < 0.01, **** *p* < 0.0001 (Between 3° to 40° day; 15° to 40° day).

Formulations stability at 4 °C and 37 °C was evaluated for 40 days and results are presented in Figure 2 and Figure 3. No significant differences were observed in PDI and Z-average for the formulations stored at either 4 °C or 37 °C, indicating stability for 40 days. The only exception was DMPC/DSPE-PEG 10% that presented a significant variation in PDI and Z-average from the 3rd to the 40th day of storage at 37 °C (Figure 3C,D). Usually, significant size variations are correlated to liposomes instability, resulting mainly from aggregation/fusion of vesicles or leakage of encapsulated material due to phospholipids degradation [46,47,48]. Nonetheless, the presence of α-tocopherol (1%) in the formulations possibly preserved the unsaturated DOPC from oxidation [49,50]. For the DMPC/DSPE-PEG 10% at 37 °C, the higher temperature might had destabilized the PEG moiety and resulted in DSPE-PEG expulsion of the bilayer with consequent alteration in its curvature. This was observed only for the DMPC-DSPE-PEG liposomes owing to the packing mismatch (14:0, DMPC; 18:1, DOPC and 18:0, DSPE-PEG).

### 3.3. L-Asparaginase Encapsulation in Liposomes

**Figure 4:**
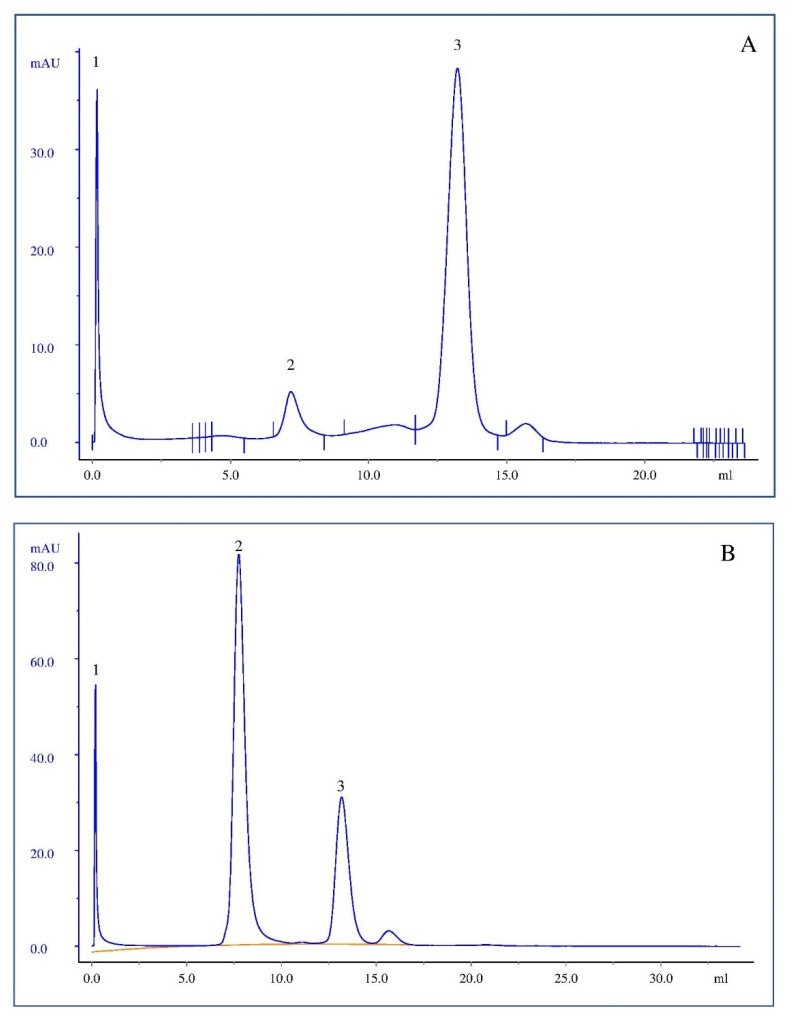
Chromatogram of Size Exclusion Chromatography. (**A**) L-asparaginase solution (5 mg/mL), *peak 1* residues flow-through; *peak 2* probably corresponds to aggregated protein and *peak 3* refers to pure and soluble ASNase solution (area corresponding to 30.65 mAU·mL). (**B**) ASNase-DOPC/DSPE-PEG 5% liposome system, *peak 1* flow-through; *peak 2* correspond to ASNase-DOPC/DSPE-PEG 5% liposomes and *peak 3* ASNase non-encapsulated (area corresponding to 27.37 mAU·mL). *n* = 4, isocratic elution with PBS (pH 7.4).

**Figure 5:**
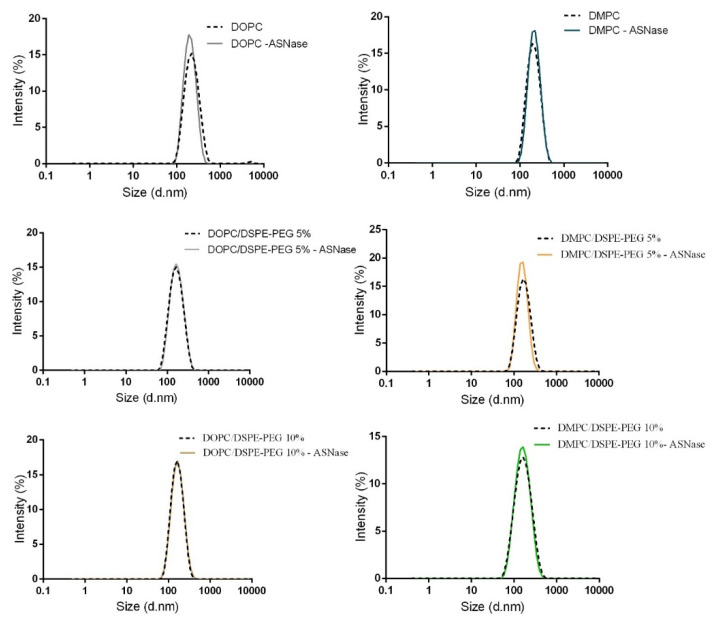
Dynamic light scattering profile of blank liposomes and ASNase-loaded liposome formulations after purification (*n* = 4).

ASNase was encapsulated in liposomes by electroporation, as previously reported for polymersomes [24]. ASNase-loaded liposomes were separated from free ASNase by Size Exclusion Chromatography (SEC). Figure 4 illustrates the chromatogram of the ASNase-DOPC/DSPE-PEG 5% formulation that represents the typical profile obtained for all ASNase-loaded liposome formulations. One can see that, as expected, the larger vesicles were eluted first and separated from the free non-encapsulated enzyme. Moreover, some degree of aggregation is observed in Figure 4A, peak 2, as expected for protein solutions. The fractions corresponding to ASNase-loaded liposomes were analyzed by DLS and, as can be seen in Figure 5, size distribution was preserved after purification by SEC. In addition, no peaks corresponding to free protein were observed; however, we should consider that the significant size difference between ASNase (~5 nm) and the liposomes (150–200 nm) can mask the possible presence of free ASNase.

**Figure 6:**
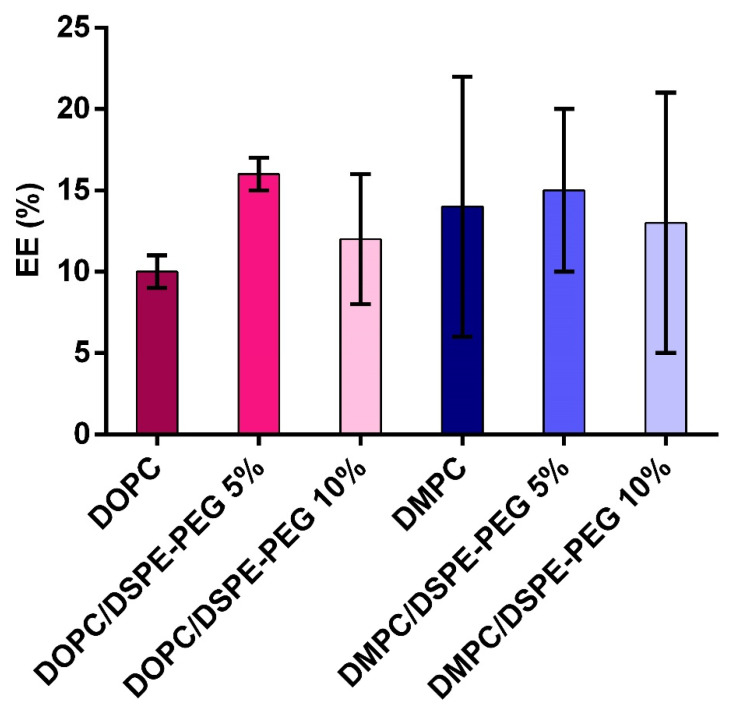
Encapsulation efficiency EE (%) of ASNase in liposomes. EE% were calculated based on the area of the chromatographic peak corresponding to the free ASNase before encapsulation and after purification by size exclusion chromatography, *n* = 4. Mean ± SD.

The peaks were integrated to calculate encapsulation efficiency (%EE) (Figure 6). The %EE values varied from 10% to 16% and no correlation was observed between %EE and the type of phospholipid or DSPE-PEG concentration (Figure 6). In fact, we expected equivalent %EE values since the liposomes were similar in size and encapsulation of ASNase is only dependent on the protein concentration in the electroporation solution. Literature reports different %EE for proteins in liposomes; BSA (Bovine Serum Albumin) encapsulation in liposomes by film hydration, for example, resulted in EE = 1% [51]. Nonetheless, our results were similar to the ones previously found by Wang and collaborators for BSA encapsulation in polymersomes [52].

A previous work in which ASNase was encapsulated in liposomes reports the rate of protein/lipid, resulting in liposomes loading capacity of 30% to 70% (%*w*/*w*) [27]. However, we believe these results are overestimated due to the use of triton X-100 to disrupt the vesicles, since this surfactant is known to interfere in the method used for protein quantification.

### 3.4. Transmission Electronic Microscopy (TEM) and Cryogenic Electron Microscopy (Cryo-EM)

**Figure 7:**
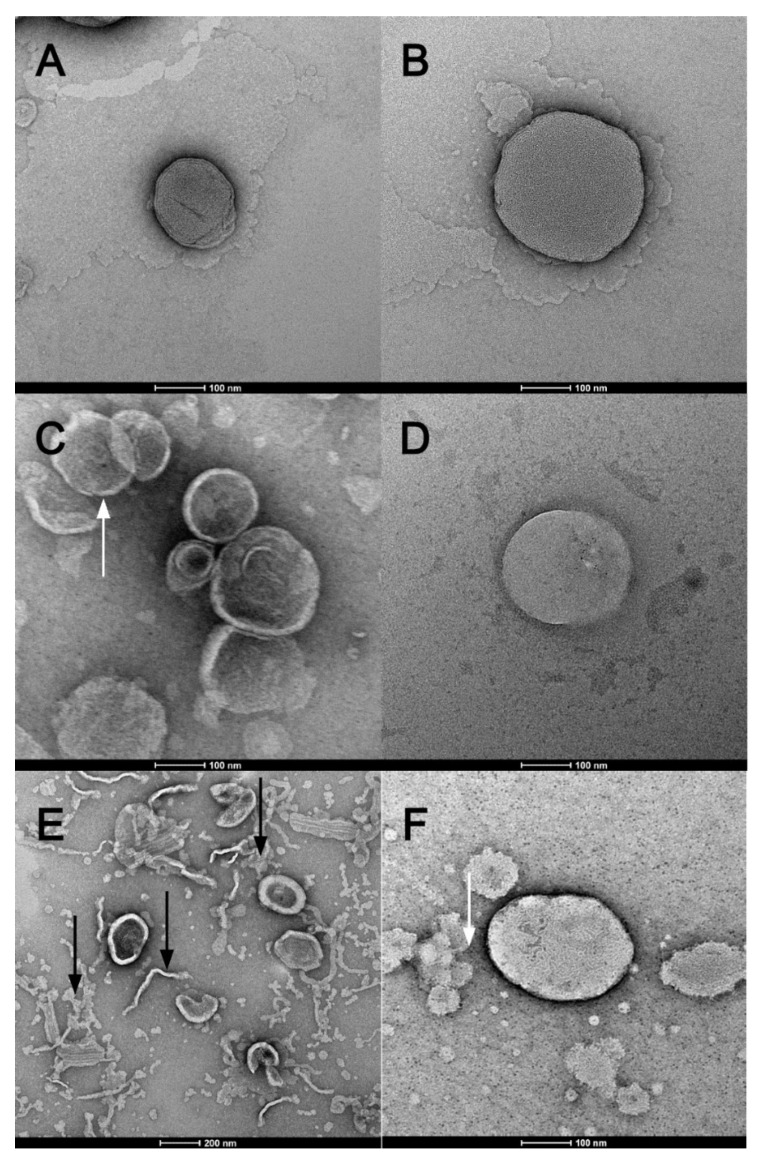
Transmission electronic microscopy (TEM). Formulations: (**A**). DMPC; (**B**). DOPC; (**C**). DMPC/DSPE-PEG 5%; (**D**). DOPC/DSPE-PEG 5%; (**E**). DMPC/DSPE-PEG 10% e (**F**). DOPC/DSPE-PEG 10%. Magnification of 25,000× (**E**) and 62,000× (**A**–**F**). The bars indicate size of 200 nm (**E**) and 100 nm others. Black arrows indicate fragments of lamellae or fragmented liposomes white arrows indicate clusters of liposomes.

TEM imagens of blank liposomes of DMPC, DMPC/DSPE-PEG 5%, DMPC/DSPE-PEG 10%, DOPC, DOPC/DSPE-PEG 5% and DOPC/DSPE-PEG 10% (Figure 7) presented deformed structures that must be fragments of lamellae or fragmented liposomes (black arrows) and clusters of liposomes (white arrows) (Figure 7C,E,F), which can be related to the disruption or damage of the bilayer during the grid´s drying or microscopy electronic vacuum [53,54].

**Figure 8:**
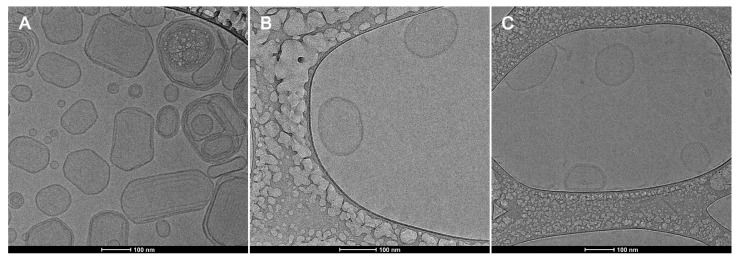
Cryogenic electron microscopy of DMPC liposomes. (**A**): DMPC formulation. (**B**): ASNase-DMPC/DSPE-PEG 10% (**C**): DMPC/DSPE-PEG 10%. Magnification of 62,000×. The bars indicate size of 100 nm.

**Figure 9:**
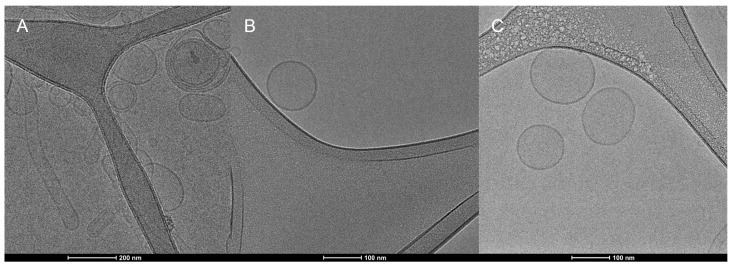
Cryogenic electron microscopy of DOPC liposomes. (**A**): DOPC formulation. (**B**): ASNase-DOPC/DSPE-PEG 5% (**C**): DOPC/DSPE-PEG 5%. Magnification of 25,000× (**A**) and 62,000× (**B**,**C**). The bars indicate size of 100 nm (**B**,**C**) and 200 nm (**A**).

Cryo-TEM images showed the liposomes shape and the lipid bilayer without deformations, PEGylated and non-PEGylated liposomes present similar morphology corresponding to unilamellar bilayers and size range of 114–145 nm (Figure 8 and Figure 9). The thickness of the liposomes bilayer was estimated based on the Cryo-TEM images and non-pegylated liposomes presented thickness of 4.8 nm ± 1 nm, similar to previous values presented in the literature [55]. Pegylated liposomes presented slightly thicker bilayers, ranging from 5.8 to 6.6 nm (Table 2). Therefore, it seems the presence of the PEGylated phospholipid resulted in an increase in the bilayer thickness, nonetheless this result needs further confirmation by a more precise technique such as Small Angle X-ray Scattering (SAXS). The presence of the enzyme and the electroporation did not influence the average size, shape and membrane thickness of the liposomes (Table 2; Figure 8 and Figure 9), corroborating with the DLS analyses (Table 1).

### 3.5. Liposomes Permeability to Asparagine

**Figure 10:**
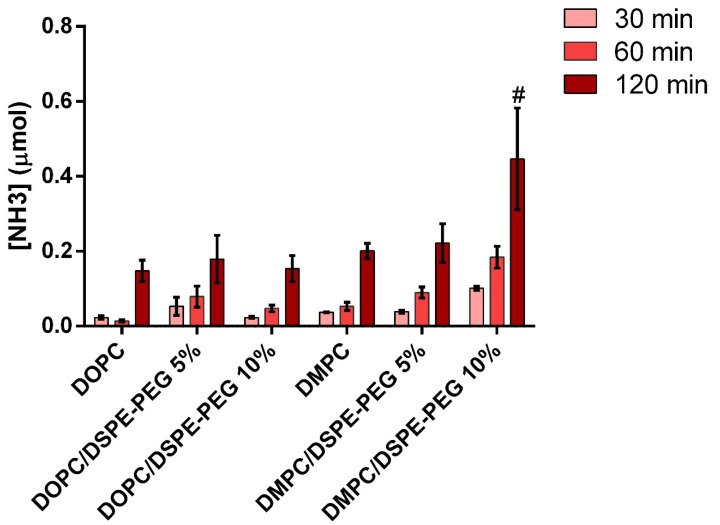
Ammonia generation in the presence of ASNase-loaded liposomes (Nessler’s assay results). # Formulation presented significant difference related to others’ formulations. *n* = 6 independent experiments. The error bars correspond to standard deviation. Two-way ANOVA, Turkey´s test, α = 0.05.

The permeability of liposomes was evaluated based on the ammonia release, a product of the L-asparagine hydrolysis catalyzed by L-asparaginase (Figure 10). As can be seen, the concentration of ammonia detected for all the systems was low (up to 0.45 M). Three hypotheses can justify these low levels of ammonia: (i) low ASNase encapsulation efficiency; (ii) low L-Asn penetration in the liposomes; and (iii) volatilization of the ammonia generated. Nonetheless, a significant increase in ammonia concentration is observed after 120 min, indicating enzyme activity and possibly vesicle permeability.

Nanocarrier permeability can be modulated by varying the phospholipids composition, since different tail length and saturation, different gel-to-fluid phase transition temperature (T_m_) as well as PEGylation result in different degrees of bilayer packing [21,22,23]. Usually, unsaturated phospholipids self-assemble in loosely packed bilayers and, therefore, we expected higher permeability for the liposomes composed of DOPC. Nonetheless, ASNase-DMPC/DSPE-PEG 10% corresponded to the highest ammonia release. We attributed this behavior to the packing mismatch between DMPC (14 methylene groups in the alkyl chain) and DSPE-PEG (18 methylene groups in the alkyl chain) that does not happen for DOPC (18 methylene groups in the alkyl chain). A packing mismatch results in a loosely packed bilayer [56]. One should bear in mind, that DOPC has one unsaturation on each of the alkyl chains.

A correlation between lipid bilayer permeability and DSPE-PEG concentration was observed, mainly for the DMPC-based formulations (Figure 10). The influence of PEGylation on permeability was previously investigated for liposomes of DPPC and DPPE-PEG 5000/DPPE-PEG 2000. According to the authors, increased permeability was observed for PEGylated vesicles in the range of DPPE-PEG transition from the mushroom (compacted) to the brush (extended) conformation [57,58]. Transition regions between the mushroom and brush conformations can generate defects in the bilayer and allow the flow of small molecules, such as L-Asn [57,58,59,60].

We must keep in mind that activity can also be related to ASNase adhered to the liposomes’ surface. We purified the systems by size-exclusion chromatography and the DLS after purification did not indicate the presence of free protein. Nonetheless, even a small number of ASNase at the surface of the vesicles could result in activity (increase in ammonia concentration).

### 3.6. In Vitro Cytotoxicity against MOLT-4 Cells

**Figure 11:**
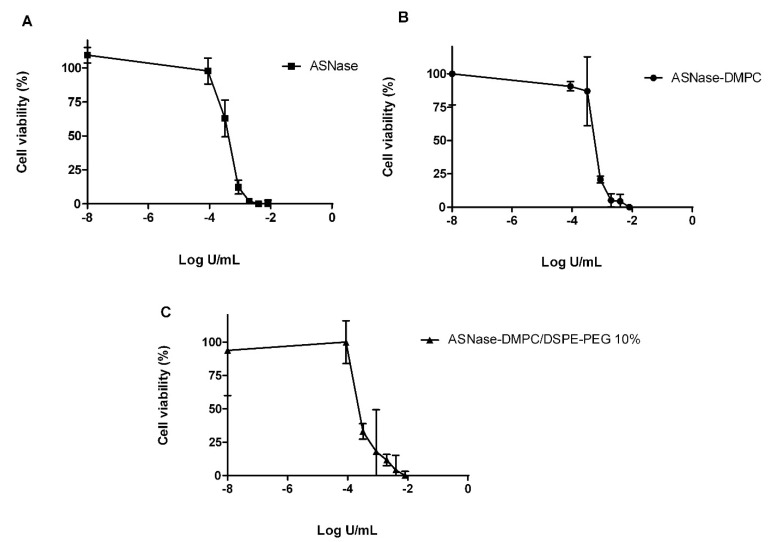
In vitro analyses of formulation. In vitro cytotoxicity of free ASNase (**A**), ASNase-DMPC (**B**) and ASNase-DMPC/DSPE-PEG 10% (**C**) formulations against MOLT-4 cells. The error bars correspond to standard deviation.

Due to the higher permeably of ASNase-DMPC/DSPE-PEG 10% to Asn, in vitro cytotoxicity against Molt-4 leukemic cell line was determined and compared to free ASNase and ASNase-DMPC as a control (Figure 11) and the IC_50_ values calculated (Table 3). As can be seen, no significant difference was detected between free ASNase and ASNase-DMPC, confirming that enzyme activity was preserved even after encapsulation and the formulation was not toxic by itself. In contrast, others authors showed a reduction in cytotoxicity of ASNase-loaded liposomes against CHO cells in comparation to free ASNase [25]. On the other hand, the IC_50_ for ASNase-DMPC/DSPE-PEG 10% was lower, highlighting the potential of this system that could allow the enzyme to circulate longer without activating the immune system.

## 4. Conclusions

We developed ASNase-loaded liposomes of DMPC or DOPC containing or not different amounts of DSPE-PEG (DOPC/DSPE-PEG 5%, DOPC/DSPE-PEG 10%, DMPC/DSPE-PEG 5%, DMPC/DSPE-PEG 10%) and encapsulated the anti-leukemic drug ASNase. Formulations were found to be capable of depleting the amino acid L-asparagine, an indication of its penetration inside the liposomes that could be working as nanoreactors. In particular, the DMPC/DSPE-PEG 10% formulation was found to be more efficient in depleting L-asparagine compared to the other systems. In vitro assay also showed that ASNase-loaded DMPC/DSPE-PEG 10% systems increased cytotoxicity against MOLT-4 leukemic cell line when compared to free ASNase and the ASNase-loaded pure DMPC liposomes. In conclusion, the system developed could be an alternative to improve the therapy with ASNase.

## Figures and Tables

**Table 1:** Dynamic light scattering and Zeta Potential profile of liposomes formulations. Formulations were analyzed for particle size based on the Z-average size and polydispersity index (PDI). Data correspond to mean ± standard deviation (*n* = 3) independent experiments, except for the zeta potential measurements (*n* = 1).

Formulation	Z-Average (nm)	PDI	Zeta Potential (mV)
DOPC	202 ± 9	0.143 ± 0.019	−3.57
DOPC/DSPE-PEG 5%	155 ± 10	0.084 ± 0.014	−3.69
DOPC/DSPE-PEG 10%	154 ± 7	0.079 ± 0.009	−5.89
DMPC	193 ± 6	0.069 ± 0.024	−4.55
DMPC/DSPE-PEG 5%	157 ± 7	0.090 ± 0.014	−3.73
DMPC/DSPE-PEG 10%	142 ± 10	0.190 ± 0.020	−2.57

**Table 2:** Evaluation of size (nm) and membrane thickness of liposomes. Average liposome diameter and bilayer thickness mean values were obtained from Cryo-EM images, using the ImageJ software. For each system, at least two vesicles were measure with 10 spots of measurement each. SD of ± 1 nm for all sample.

Formulations	Average Size (nm)	Bilayer Thickness Average (nm)
DOPC	114	4.7
DOPC/DSPE-PEG 5%	140	6.0
DMPC	157	4.9
DMPC/DSPE-PEG 10%	126	5.8
ASNase-DOPC/DSPE-PEG 5%	148	5.8
ASNase-DMPC/DSPE-PEG 10%	145	6.6

**Table 3:** Values of IC_50_ of pure ASNase, ASNase-DMPC and ASNase-DMPC/DSPE-PEG 10% based on cytotoxicity assays against Molt-4 cell line. Mean ± SD, *n* = 2.

Formulations	IC_50_ (U/mL)
ASNase	0.000376 ± 0.000027
ASNase-DMPC	0.000548 ± 0.000044
ASNase-DMPC/DSPE-PEG 10%	0.000267 ± 0.000029

## Data Availability

Not applicable.

## Supplementary Materials

The following are available online at https://www.mdpi.com/article/10.3390/pharmaceutics14091819/s1, Figure S1. Dynamic Light Scattering graphs. Size distribution by intensity and Raw correlation data of DOPC formulations. Figure S2. Dynamic Light Scattering graphs. Size distribution by intensity and Raw correlation data of DOPC/DSPE-PEG 5% formulations. Figure S3. Dynamic Light Scattering graphs. Size distribution by intensity and Raw correlation data of DOPC/DSPE-PEG 10% formulations. Figure S4. Dynamic Light Scattering graphs. Size distribution by intensity and Raw correlation data of DMPC formulations. Figure S5. Dynamic Light Scattering graphs. Size distribution by intensity and Raw correlation data of DMPC/DSPE-PEG 5% formulations. Figure S6. Dynamic Light Scattering graphs. Size distribution by intensity and Raw correlation data of DMPC/DSPE-PEG 10% formulations. Figure S7. Dynamic Light Scattering graphs. Size distribution by number and Raw correlation data of ASNase-DOPC. Figure S8. Dynamic Light Scattering graphs. Size distribution by number and Raw correlation data of ASNase-DOPC/DSPE-PEG 5%. Figure S9. Dynamic Light Scattering graphs. Size distribution by number and Raw correlation data of ASNase-DOPC/DSPE-PEG 10%. Figure S10. Dynamic Light Scattering graphs. Size distribution by number and Raw correlation data of ASNase-DMPC. Figure S11. Dynamic Light Scattering graphs. Size distribution by number and Raw correlation data of ASNase-DMPC/DSPE-PEG 5%. Figure S12. Dynamic Light Scattering graphs. Size distribution by number and Raw correlation data of ASNase-DMPC/DSPE-PEG 10%.
