# Supplementary material for: Peg-Grafted Liposomes for L-Asparaginase Encapsulation
Source: Pharmaceutics. 2022 Aug 29;14(9):1819. doi: 10.3390/pharmaceutics14091819 (PMC9503594; doi:10.3390/pharmaceutics14091819)
Supplement: Supplementary file 1 [file pharmaceutics-14-01819-s001.zip › pharmaceutics-1861747-supplementary.pdf]

Supplementary Files

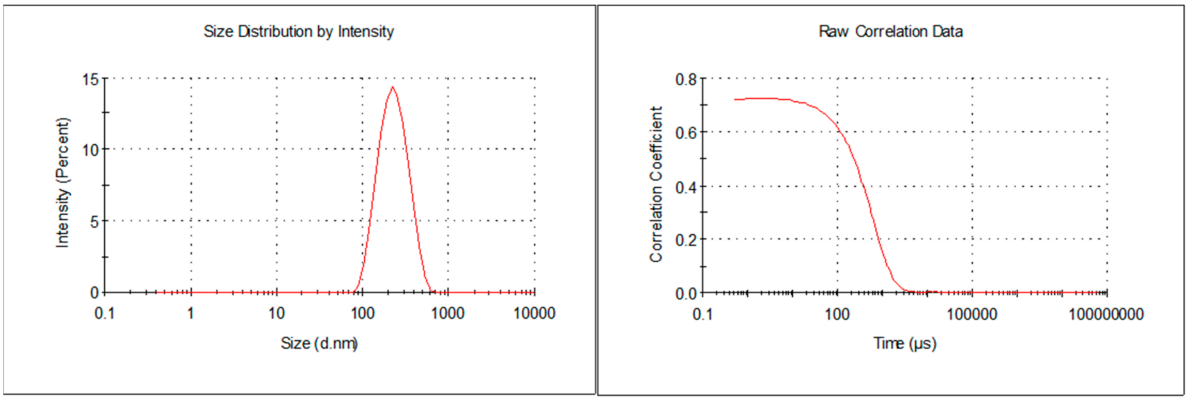

Figure S1. Dynamic Light Scattering graphs. Size distribution by intensity and Raw correlation data of DOPC formulations.

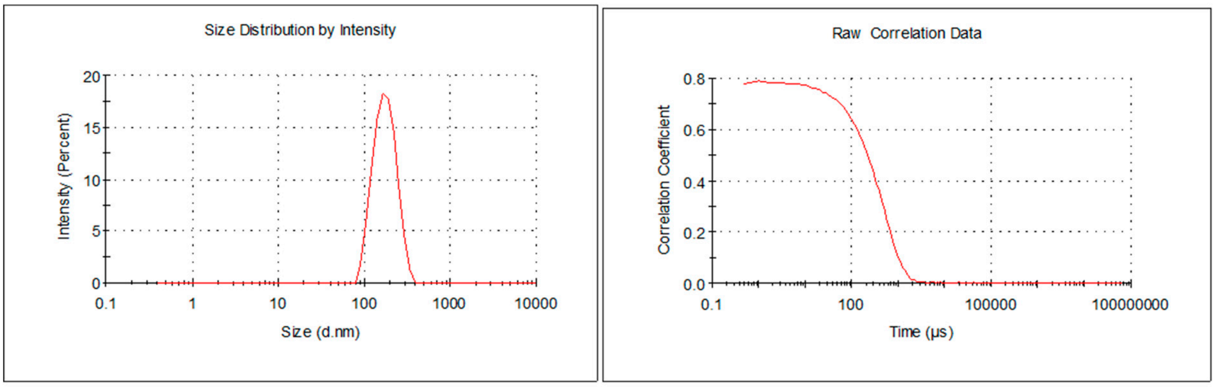

Figure S2. Dynamic Light Scattering graphs. Size distribution by intensity and Raw correlation data of DOPC/DSPE-PEG 5% formulations.

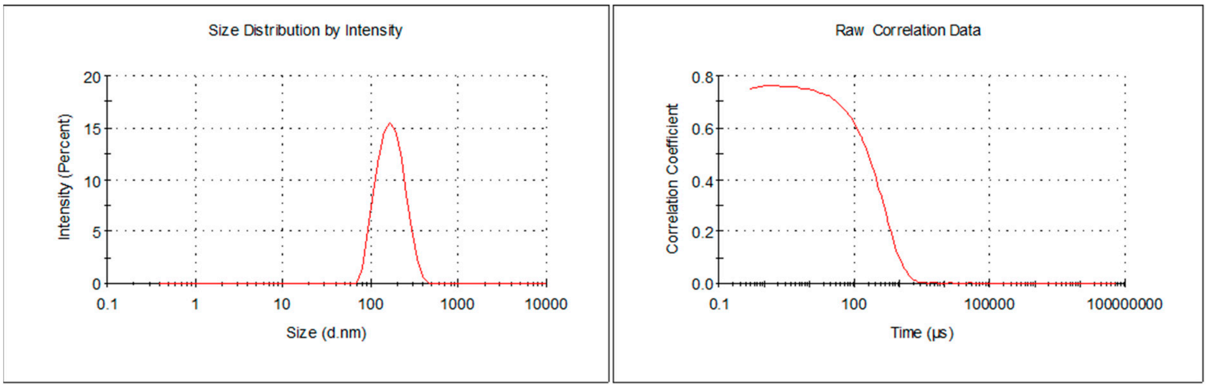

Figure S3. Dynamic Light Scattering graphs. Size distribution by intensity and Raw correlation data of DOPC/DSPE-PEG 10% formulations.

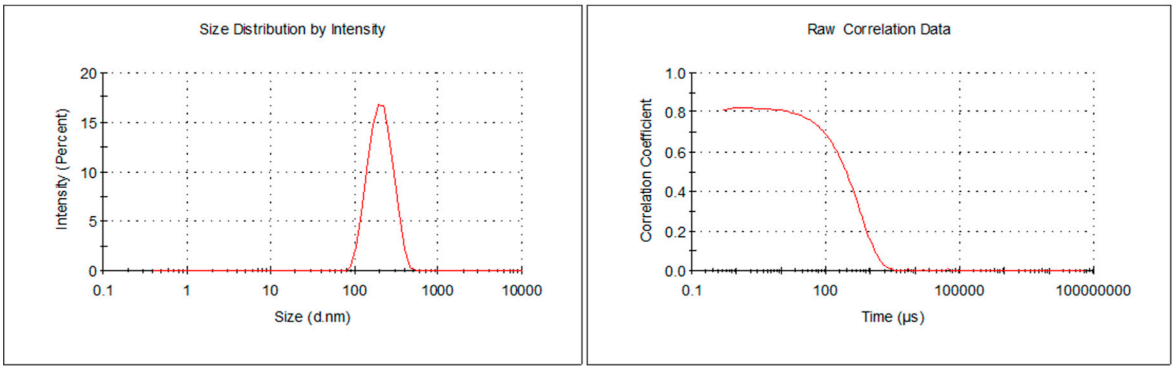

Figure S4. Dynamic Light Scattering graphs. Size distribution by intensity and Raw correlation data of DMPC formulations.

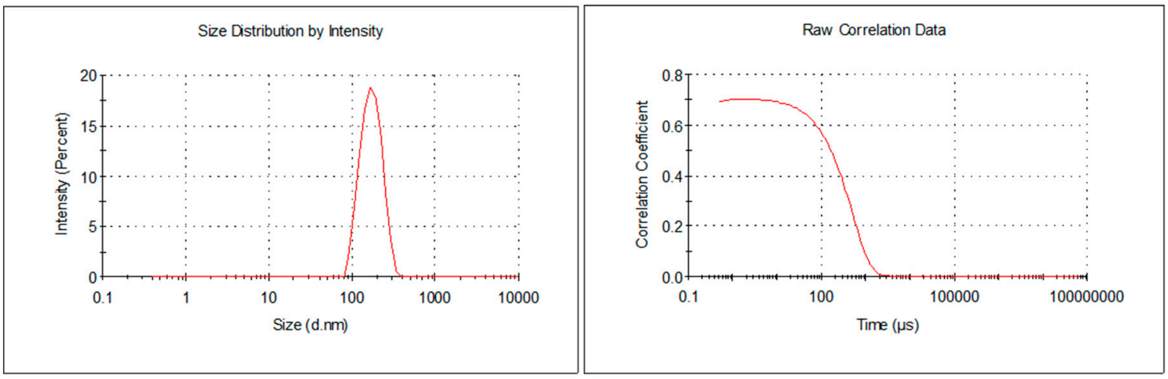

Figure S5. Dynamic Light Scattering graphs. Size distribution by intensity and Raw correlation data of DMPC/DSPE-PEG 5% formulations.

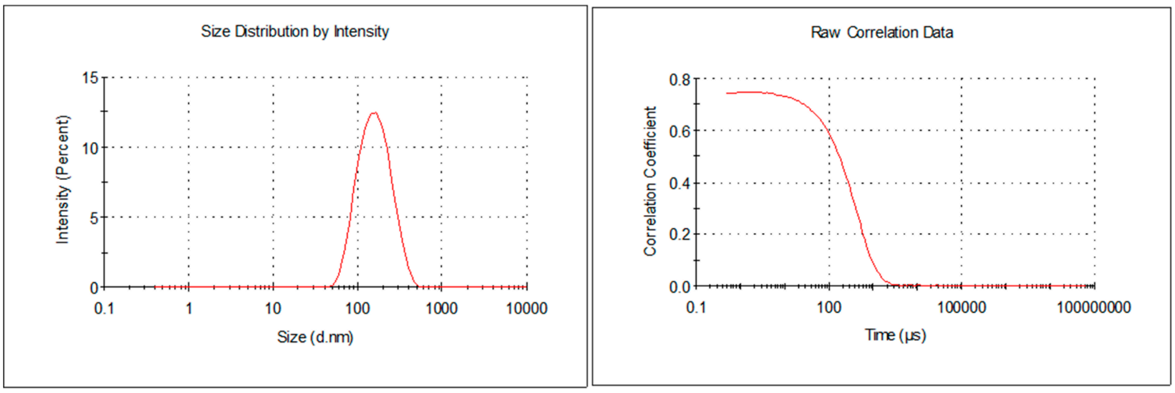

Figure S6. Dynamic Light Scattering graphs. Size distribution by intensity and Raw correlation data of DMPC/DSPE-PEG 10% formulations.

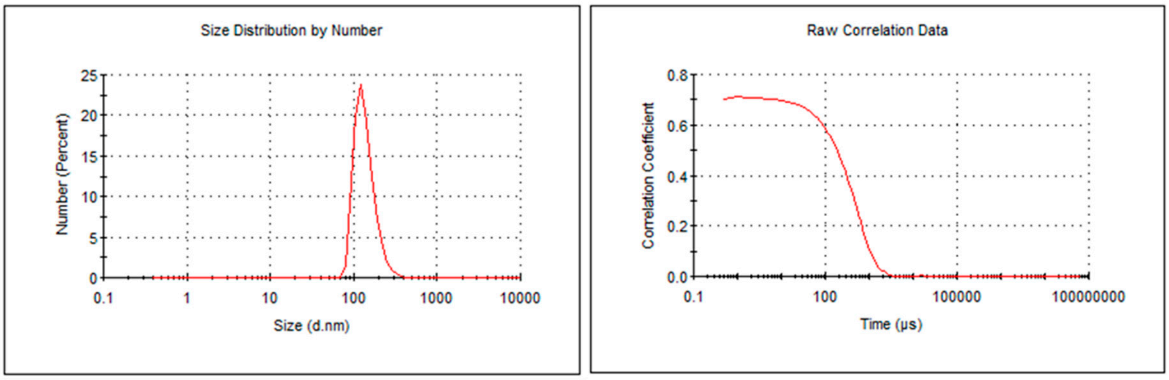

Figure S7. Dynamic Light Scattering graphs. Size distribution by number and Raw correlation data of ASNase-DOPC.

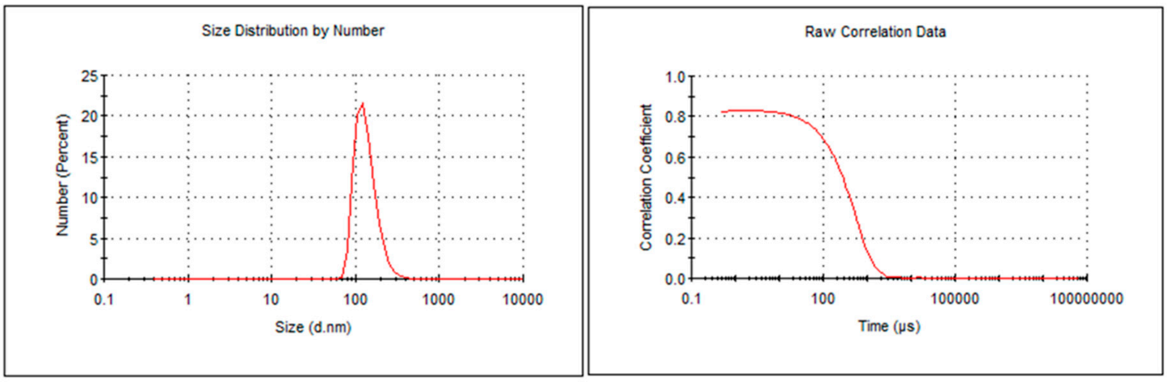

Figure S8. Dynamic Light Scattering graphs. Size distribution by number and Raw correlation data of ASNase-DOPC/DSPE-PEG 5%.

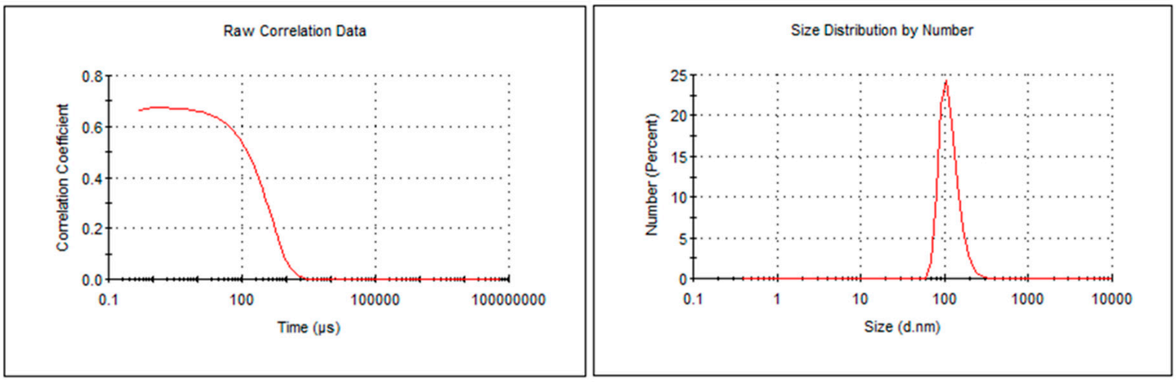

Figure S9. Dynamic Light Scattering graphs. Size distribution by number and Raw correlation data of ASNase-DOPC/DSPE-PEG 10%.

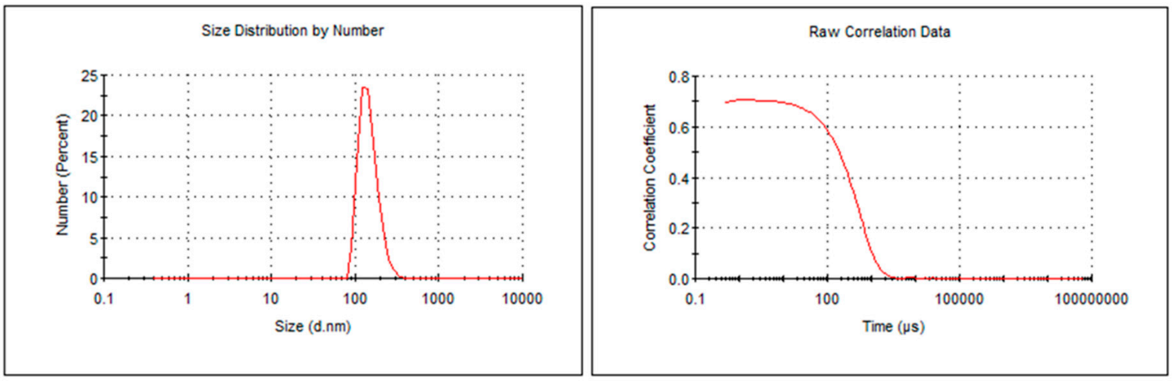

Figure S10. Dynamic Light Scattering graphs. Size distribution by number and Raw correlation data of ASNase-DMPC.

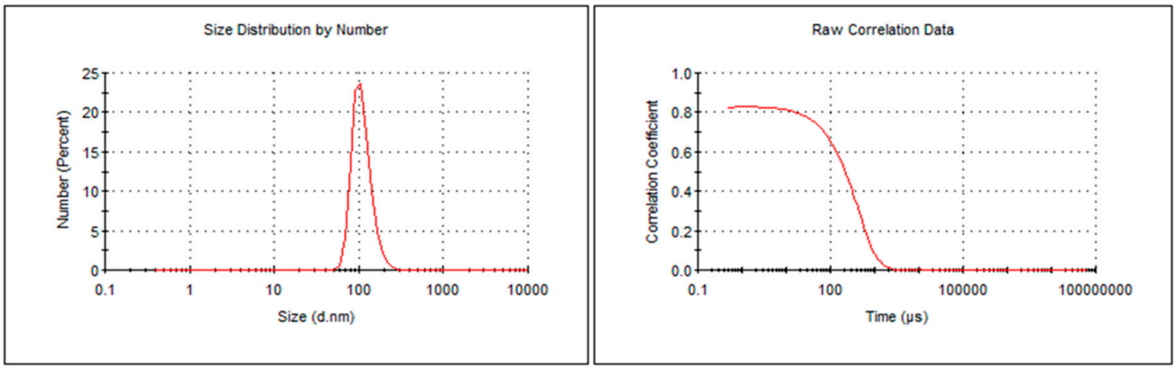

Figure S11. Dynamic Light Scattering graphs. Size distribution by number and Raw correlation data of ASNase-DMPC/DSPE-PEG 5%.

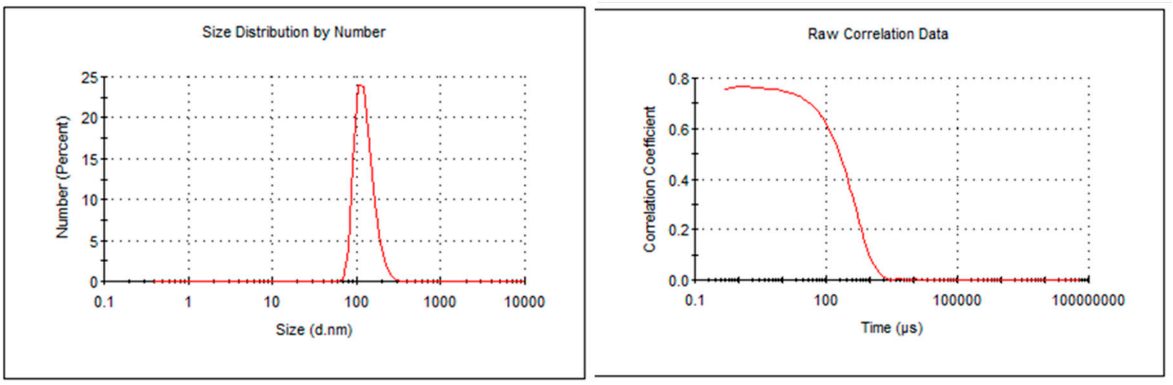

Figure S12. Dynamic Light Scattering graphs. Size distribution by number and Raw correlation data of ASNase-DMPC/DSPE-PEG 10%.
